# An Assessment of Anki Flashcards Use in Comparison to Alternative Study Methodologies in First Year Graduate Entry Medical Students

**DOI:** 10.1007/s40670-025-02504-7

**Published:** 2025-10-06

**Authors:** Colleen Haughey, Sten Kajitani, Maria Buckley, Mawadda Mohamed, Mark Rae, Elizabeth Brint

**Affiliations:** School of Medicine, University College Cork, Cork, Ireland

**Keywords:** Anki, Spaced repetition learning, Assessment outcome

## Abstract

First year graduate entry to medicine (GEM) students require effective personalised study techniques in order to learn significant amounts of factual information. This study analysed learning methodologies utilised by a cohort of first year GEM students and their perception of their utility, particularly of the spaced-repetition learning app, Anki. Furthermore, our study compared assessment performance with study methodology utilised by this cohort of students. In total, 53 students participated in some aspect of the study, with 43 students completing all three components: a pre-module test to assess baseline knowledge of module content, a post-module test, and a survey questionnaire. Our survey revealed that students utilised a diverse range of learning and study methodologies. In terms of satisfaction rating, students positively perceived preparedness for exams and time management, irrespective of the study methodology used. While 80% of study participants reported using Anki, the number of Anki cards used and its daily usage varied significantly among users. Overall, there was no statistically significant benefit for Anki usage in terms of performance outcome compared to those who did not use Anki when the module was considered as a whole. However, we did find it to be of significant benefit to exam performance in two categories: (1) those who used Anki extensively compared to those who did not engage consistently, and (2) a significant correlation between Anki usage and physiology exam performance was identified.

**Supplementary Information:**

The online version contains supplementary material available at 10.1007/s40670-025-02504-7.

## Introduction

First year medical students are required to learn and retain significant volumes of course material delivered at pace [1, 2]. This is particularly true of the accelerated graduate entry to medicine (GEM) programmes, common in Ireland and the UK, which are open to students who have previously completed an undergraduate degree and can be completed in 4 years [3]. As such, the issue of content understanding and retention is significant for these students, particularly those from a non-biomedical sciences background. Exacerbating the need for vast information understanding, examination formats are often multiple-choice question (MCQ) based, such as the high-stakes United States Medical Licensing Examination (USMLE), which requires a high degree of recall by the students [4, 5].

Advances in artificial intelligence, online learning tools, and regular recording of didactic lectures have provided students with a myriad of options for developing a toolkit with which to enhance their learning and information retention. One such methodology is spaced-repetition learning, relying on regular, repeated learning sessions. Several studies have demonstrated the efficacy of spaced repetition, also known as expanding retrieval practice, as a learning tool [2, 6, 7]. For example, a study by Dobson demonstrated that participants who underwent any form of active recall/retrieval practice performed approximately twice as well as controls [8]. Therefore, electronic-based spaced repetition aims to reduce the cognitive load of rote memorisation [9]. Spaced repetition is often conducted in conjunction with flashcards, which are a method in which a prompt is displayed on one side of the card and the answer on the opposite [6, 10, 11]. Multiple platforms now exist that enable students to combine the use of digital flashcards with spaced repetition [4, 10, 12]. Anki is a popular spaced repetition platform that is utilised by medical students globally to support learning associated with medical curricula [12–17]. As per a scoping review of existing research on electronic flashcards, 78% of studies focused on utilisation of techniques, but only 60% on score outcome [18]. Some studies with a focus on Anki usage and score improvement have reported that students with more consistent Anki usage achieve higher USMLE Step 1 scores and may have a general positive correlation with Anki use and examination scores [7, 13, 19].

In line with international trends, students in the first year GEM degree programme in University College Cork (UCC), Ireland, are increasingly using Anki [7, 19, 20] with an Anki bank comprising 15,000 flashcards specific to the first year course material created by UCC GEM students over the last few years. The aim of this study, therefore, was to investigate study methodologies used by first year GEM students in a multi-disciplinary integrated module comprising teaching across four disciplines (anatomy, physiology, pharmacology, pathology). Given the multifaceted nature of medical student learning strategies, we employed a mixed-methods approach to capture both quantitative outcomes (test performance) and qualitative insights (student perceptions) regarding study methodologies. We hypothesised that increased Anki usage would correlate with greater performance improvement. Where students reported using Anki, we assessed the extent of their use of the Anki app using data uploaded by students indicating daily, weekly, and total flashcards viewed and compared usage with their assessment performance. The combination of quantitative (pre- and post-module test scores) and qualitative (survey responses, including Likert-scale and free-text items) data allowed us to not only evaluate measurable learning outcomes but also to explore students’ perceptions and experiences with different study tools. This design was particularly suitable for capturing both the effectiveness and perceived utility of Anki compared to other learning methodologies, aligning with our aim to understand how study strategies influence both performance and learner satisfaction.

## Methods

### Context and Participants

The study was approved by the UCC Social Research Ethics Committee. The study took place during semester two of year 1 of the UCC GEM programme. Students entering the GEM programme at UCC must possess at least one undergraduate degree, at a minimum grade of a second-class honours, grade 1, or a 3.3 grade point average. Content covered material delivered in the “Fundamentals of Medicine II” module. Teaching on this 11-week module relates to the cardiovascular, respiratory, renal, and genitourinary systems and includes integrated structure and function, pathological basis, clinical presentation, and pharmacological management of these systems and diseases affecting them. As such, this multidisciplinary module comprises equal amounts of teaching from four life sciences disciplines: anatomy, pharmacology, physiology, and pathology. The pedagogies utilised in this module included lectures, tutorials, gross anatomy dissections, and small group case-based learning activities. A sample size calculation indicated that the study required a minimum of 16 participants to have sufficient power to predict increased performance on exam scores, with a type 1 error rate of 5%, absolute error of 5%, and standard deviation of exam scores of 10% [13, 19, 21].

In total, 53 of the 80-year 1 GEM enrolled students were recruited to participate in the study, all of whom completed the survey. Of those, 43 participants also completed all components (pre-test, post-test, and survey). Analyses involving correlations with assessment performance were therefore limited to the 43 students who completed all components, while survey-based analyses include data from all 53 respondents. The 10 students who completed the survey but not both assessments showed no notable differences in terms of gender, biomedical background, or reported study methods as compared to the 43 who completed all components.

### Study Design

Participants were required to undertake a pre-module test in the first week of the module to establish a baseline of their prior knowledge of the module content. The pre-module test comprised 40 multiple-choice questions in a best of five “single best item” (SBI) format, where a question stem is answered by selecting one of up to five possible answers. The 40 questions comprised 10 each from the four contributing disciplines. On the final day of the module, participants completed the study survey and then undertook a post-module test of an additional 40 questions, of similar difficulty to the pre-module test, as determined by the question contributors. The difference between the pre-module and post-module test was then calculated and aligned with survey results. All results and survey answers were anonymised and matched by a unique identifier code assigned to each participant. Qualitative data was included in the survey to quantify students’ experiences with their chosen study techniques. Free-text responses related to Anki usage were reviewed by two authors and grouped into broad descriptive categories of positive, negative, or mixed perceptions. Comments were independently reviewed, and any differences in classification were resolved through discussion.

### Data Collection

Pre- and post-module tests were conducted on the Socrative platform, which enabled participants to enter a consistent anonymous identifier [22]. This ensured that individual pre- and post-test scores could be accurately matched while maintaining participant confidentiality, thereby enhancing the integrity and traceability of the data. The survey instrument consisted of 21 items divided into four sections: (1) demographics, (2) study methodologies used across the module, (3) Anki use, and (4) perceptions of the utility of study methodologies used across the module. The question formats were mixed and included questions where students could select multiple options, Likert scale, and free text. The Likert scales consisted of five options: 1, strongly disagree; 2, disagree; 3, neither agree nor disagree; 4, agree; 5, strongly agree. The questionnaire is available in Appendix 1.

### Data Analysis

Of the 53 participants who completed the survey, 43 also completed both the pre- and post-module assessments and were included in all analyses involving test performance. Survey-only analyses (e.g. perception of study techniques) were conducted using the full set of 53 respondents. Where subgroups (e.g. Anki preference or extent of use) are presented, the sample sizes reflect the number of valid responses in that category and are indicated in each table or figure caption.

For the analysis of data relating to the module SBIs, we calculated the percentage difference between the pre- and post-test percentages as a percentage for the overall test, as well as in each discipline. The result was denoted as “% increase in test score”.

Four groups were created to represent participants’ interaction with Anki: (1) those who had not engaged at all with Anki (*n* = 11); (2) those who had viewed 1–1000 cards at the time of the survey (*n* = 11); (3) those who had viewed 1001–3000 cards (*n* = 10); and (4) those who had viewed 3000–5000 cards (*n* = 11). These Anki usage categories were selected to create approximately equally sized groups based on the distribution of total flashcards reviewed among participants to facilitate valid statistical comparisons. Analysis of the possible correlation between students’ frequency of Anki usage and “% increase in test score” was also conducted.

To analyse preference of study methodologies, three groups were identified: (1) those indicating a primary preference for Anki (*n* = 15); (2) those indicating a primary preference for creating written notes (*n* = 19); and (3) those indicating any other primary preference, such as practice questions from lecturers, rewatching recorded lectures, or third-party resources such as UWorld and Amboss (*n* = 9).

Students’ first preference study method was correlated with “% increase in test score”. Data were entered into GraphPad Prism for statistical analyses using either chi-squared test or one-way ANOVA and Dunnett’s post-test as appropriate. Values of *P* < 0.05 were considered statistically significant (denoted as * on graph).

## Results

### Learning and Study Methodologies Utilised

**Fig. 1:**
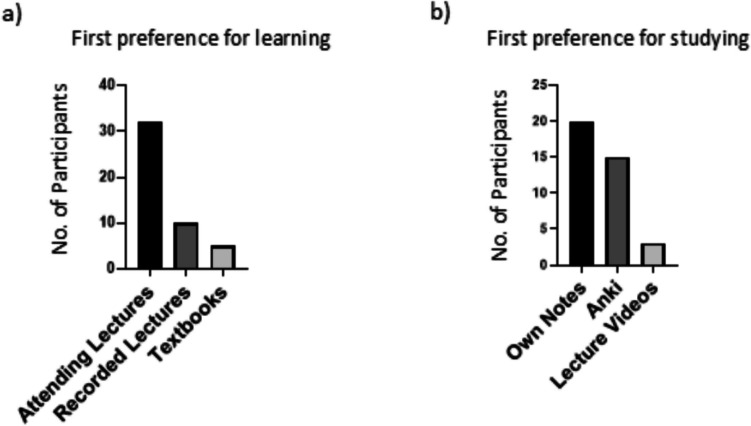
First preference methodologies for learning and studying/revising course material. Participants chose from a list of options in the survey (see Appendix 1) to identify their preferences for learning (**a**) and studying (**b**) new material. Only participants’ first preference choice is shown

Students were initially asked to rank their preferred tools for learning (first exposure to material) course material, with options including attending lectures, reading textbooks, using the spaced repetition app Anki, viewing recorded lectures, YouTube, and so on. As shown in Fig. 1a, the most popular student choice for learning new material was attending lectures (60.4%), with watching recorded lectures (18.9%) being selected as the second preference in this category. One student out of 53 identified Anki as a first preference for learning new material. Students were also asked to rank their preference for studying (revising and reviewing material) course material, with a similar list of options to those for the previous question. As shown in Fig. 1b, the most popular first preference option for revising material was students creating their own revision notes (37.7%), followed by Anki as a second preference (28.3%).

### Students’ Satisfaction Concerning Study Methodologies Utilised

**Fig. 2:**
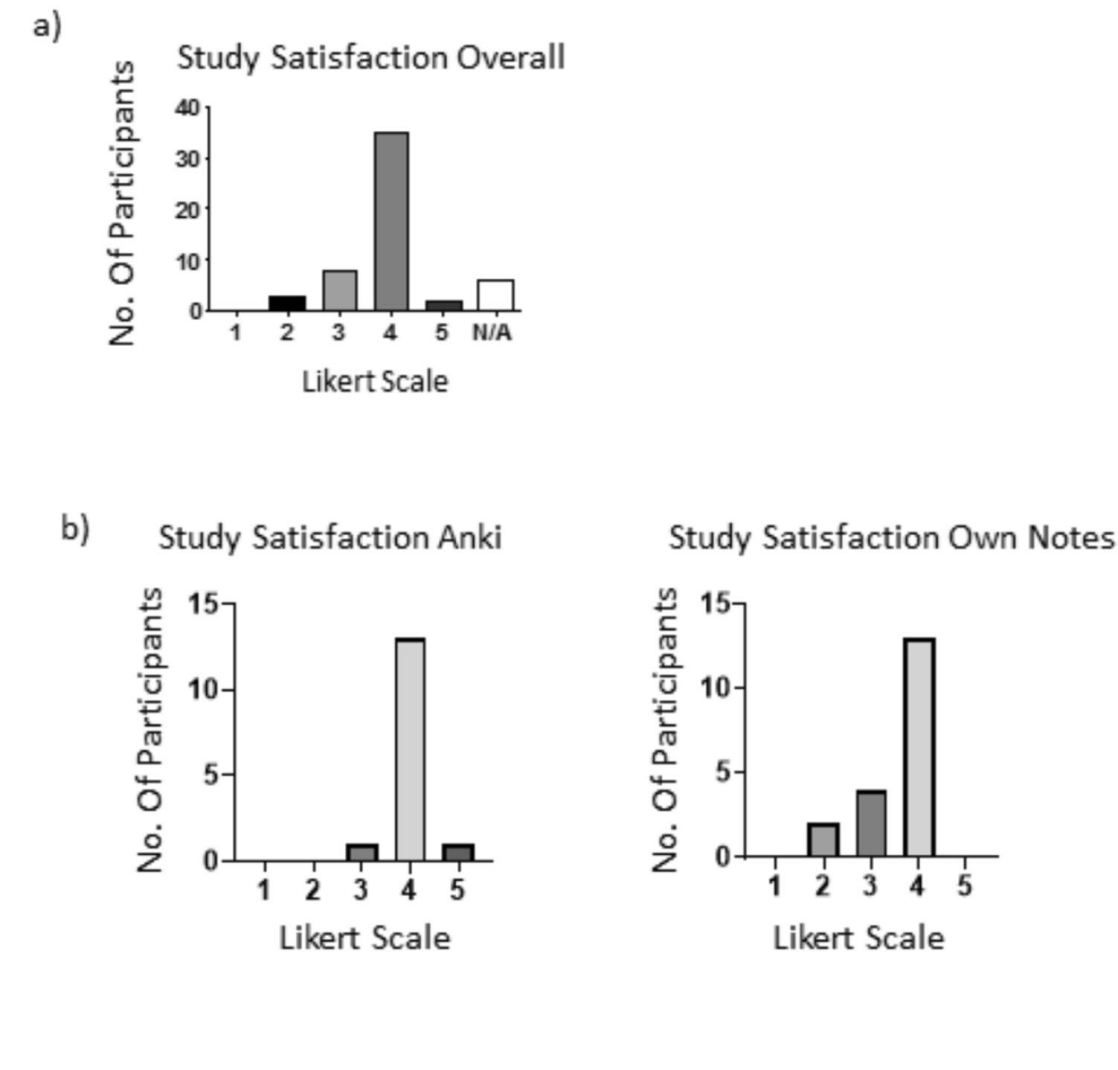
Satisfaction with study methodologies. Participants were asked to rate the statement “I was satisfied with the study techniques I employed this term” with **a**) showing study satisfaction for all particpants and **b**) study satisfaction for those who highlighted either Anki or making their won notes as their study methodology of preference. Responses were rated on a Likert scale of 1–5 where 1 = strongly disagree and 5 = strongly agree

When students were asked to indicate their degree of satisfaction with their study techniques in response to the question “I was satisfied with the study techniques I employed this term” on a Likert scale of 1–5 where 1 = strongly disagree and 5 = strongly agree, their responses were positive with 75% ranking their satisfaction level as either 3 or above out of 5 (Fig. 2a). This indicates that, although students utilised a varied array of study/revision methodologies (Fig. 1b), they were predominantly content with whatever method they chose.

As the majority of students selected either “Creating your own class notes” or “Anki” as their first preference for study/revision technique (Fig. 1b), the responses to these two study methodologies were subsequently disaggregated in order to assess students’ satisfaction with each of them. These data show a similar trend to that shown in Fig. 2a, with 86% of Anki users and 70% of Own Notes users rating their satisfaction as 4 (Fig. 2b).

### Students’ Perception of Preparedness for Exams, Knowledge Retention, and Time Management, as Related to Their Preferred Study Methodology

Students were asked to indicate whether they felt their study techniques had improved their knowledge and retention of taught material, whether they felt prepared for the end of module exam using their preferred study methodologies, and whether their study methodologies allowed them to manage their time efficiently. Responses were disaggregated into groups who either used Anki or self-created study notes. Only participants who indicated “Anki” or “Creating own notes” as their first-preference study method (*n* = 35) were included in this comparison. There were no significant differences noted between the groups who used Anki and those who used their own notes in terms of overall perception of knowledge retention, preparedness for exams, and time management, as shown in Table 1.

**Table 1:** Comparison of students’ perception between Anki use and creation of own notes. *P* value calculated using chi-squared test to compare those who voted 1, 2, 3 with those who voted 4, 5

	% of students scoring a 4 or 5 (i.e. 4 = agree, 5 = strongly agree)		*P* value
	Anki users (*n* = 15)	Own notes (*n* = 20)	
“My study techniques improved my **knowledge and retention** of course content during this study period”	60% (9/15)	85% (17/20)	0.199
“I felt **adequately prepared** for the end of term continuous assessment by using my study techniques”	60% (9/15)	30% (6/20)	0.153
“My study techniques allowed me to **manage my time in an efficient manner**”	60% (9/15)	60% (12/20)	1.0

While 81% of survey respondents reported using the Anki flashcard spaced-repetition learning app, of those, only half reviewed more than 30 cards daily. Therefore, four groups of Anki users were defined based on how much they had engaged with Anki at the time of the survey as described in the “Methods” section. As we were particularly interested in how Anki use might help with knowledge retention, exam preparedness, and time management, we disaggregated the above data in Table 1 into those responses from students who either (1) had never used Anki or had reviewed between 1 and 1000 cards at the time of the survey (Anki groups 1 and 2; *n* = 22), or (2) those who used it more extensively by reviewing between 1001 and 5000 cards at the time of the survey (Anki groups 3 and 4; *n* = 21), and compared responses between these two groups to the above questions (Table 2).

**Table 2:** Comparison of students’ perception between those who use Anki either not at all/slightly (groups 1 and 2) and those who used it more extensively (groups 3 and 4). *P* value calculated using chi-squared test to compare those who voted 1, 2, 3 with those who voted 4, 5

	% of students scoring a 4 or 5 (i.e. 4 = agree, 5 = strongly agree)		*P* value
	Anki users groups 1 and 2 (*n* = 22)	Anki users groups 3 and 4 (*n* = 21)	
“My study techniques improved my **knowledge and retention** of course content during this study period”	68% (15/22)	86% (18/21)	0.318
“I felt **adequately prepared** for the end of term continuous assessment by using my study techniques”	41% (9/22)	52% (11/21)	0.654
“My study techniques allowed me to **manage my time in an efficient manner**”	55% (12/22)	66% (14/21)	0.617

While no statistical significance was identified between the response to these survey questions between those in groups 1 and 2, metrics showed an increasing trend in positive attitudes/perspectives for those in group 2. For example, 81% of group 2 respondents reported that they felt that their study method helped with knowledge retention compared to 68% for group 1, 50% felt that they adequately prepared for their end-of-year examinations compared to 41% in group 1, and 64% reporting that they felt that this study method helped them to use their time efficiently compared to 55% in group 1.

### Assessment Outcomes

A key component of this study was to obtain quantitative data comparing preferred study methodology to assessment outcome. As such, students engaged in two examinations involving multiple-choice questions (10 each from the four disciplines subjects that comprise the module), with the first test being sat at the beginning of the module (pre-test) and the second being taken at the end of the module (post-test). The overall average score of participants for the pre-test was 33% (± 8.47) reflecting the fact that the questions related to material students had not yet been formally taught in the module. Overall, we observed an increase in student scores of 22% (± 12.09) between the pre- and post-tests (overall average score for post-test = 55% ± 9.78). This improvement in overall performance likely reflects the 11 weeks they spent attending lectures, tutorials, and all other teaching sessions in the module, and engaging in their own learning activities related to the same. A full breakdown of average scores of both tests is shown in Table 3, both overall and by individual discipline. Once the average score of the pre-test was subtracted from the average score of the post-test, it was found that there was a large range in the average percentage increase in test score, depending on the discipline. For example, there was only a 1% increase in average scores for pharmacology, compared to a 31.1% average increase for anatomy, reflecting some differences in students’ understanding of the material and the challenge level of the questions.

**Table 3:** Average scores in pre- and post-test

	Mean % ± SD		Mean % ± SD
**Pre-test all**	33 ± 8.47	**Post-test all**	55 ± 9.78
**Pre-test pathology**	32.9 ± 15.15	**Post-test pathology**	54.8 ± 16.77
**Pre-test anatomy**	44.4 ± 10.61	**Post-test anatomy**	75.5 ± 12.1
**Pre-test pharmacology**	40.6 ± 14.7	**Post-test pharmacology**	41.6 ± 18.8
**Pre-test physiology**	30.6 ± 11.9	**Post-test physiology**	51 ± 18.1

### Analysis of Assessment Outcomes Based on Study Methodology Preference

**Fig. 3:**
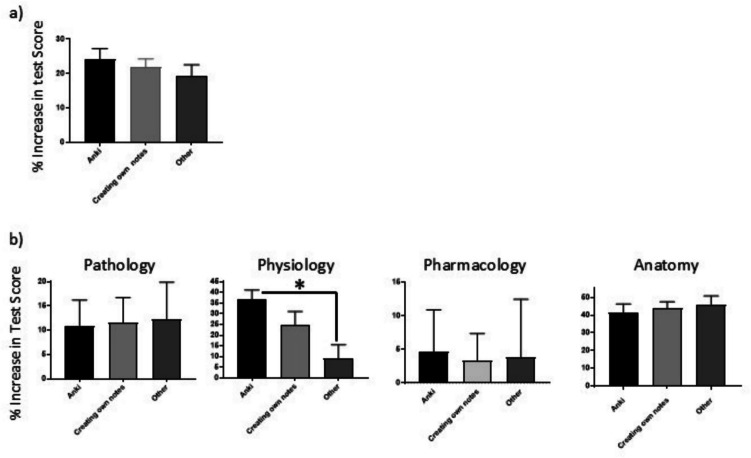
Comparison of preferred study methodology by “% increase in test score”. The difference between the % score obtained in the pre-test was subtracted from the % score in the post-test to generate the “% increase in test score”. The average of the % increase in test score across all participants was calculated and compared to study methodology of choice either for the overall test (**a**) or by individual disciplinary questions (**b**). Data analysed statistically by one-way ANOVA

As we were interested in determining whether those students that used the Anki flashcard database as a learning tool more extensively/consistently (groups 3 and 4) performed better or worse in the assessment as compared to those who used more traditional study methodologies, the “% increase in test score” was further analysed by correlating it with students’ identified first preference revision methods. For this analysis, three groups were identified: (1) those participants who indicated their primary preference of revision method was Anki (*n* = 15); (2) those whose primary preference was creating class notes (*n* = 19); and (3) those who indicated a third option including practice questions from lecturers, rewatching recorded lectures, and third-party resources (e.g. UWorld, Amboss) (*n* = 9). There were no statistically significant differences identified between any of these three groups in terms of the average percentage increase in test score (Fig. 3a). However, when these groups were further broken down into individual disciplines, it was noted that there was a significant reduction between the Anki group and the “Other” group for physiology (*p* = 0.0015). With respect to the physiology-related questions, the average percentage increase in test score for the Anki group was 35% (± 10.2), whereas the average increase in test score for the “Other” group was 6% (± 12.1) (*P* = 0.0231). This downward trend was observed between the Anki group and the class notes group, who achieved an average percentage increase in test score of 25% (± 16.2) (Fig. 3b). These data indicate that Anki is most beneficial for the study of physiology in this module.

### Analysis of Assessment Outcome Based on Extent of Anki Usage

**Fig. 4:**
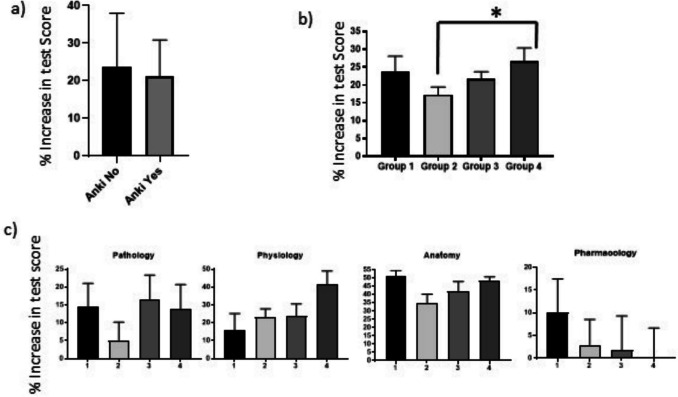
A significant difference in percentage increase in test score was identified based on the extent of Anki usage. Data shown are a comparison of percentage increase in test score between students who used Anki and those who did not (**a**); a comparison of percentage increase in test score stratified by level of Anki usage (group 1: no usage; group 2: 1–1000 cards; group 3: 1001–3000 cards; group 4: 3001–5000 cards) (**b**); a breakdown of percentage increase in test score across the four individual disciplines (anatomy, physiology, pharmacology, and pathology) for each Anki usage group (**c**). Bars represent group means and error bars indicate standard deviation. Data analysed using one-way ANOVA

As stated previously, while 81% of survey respondents reported using the Anki flashcard spaced-repetition learning app, of those, only half reviewed more than 30 cards daily. Therefore, we next conducted an analysis of the difference in the “% increase in test score” between the Anki and other groups. Initially, participants were divided into two groups; those who stated that they did not use Anki at all (*n* = 11), and those who had used Anki (to any extent) (*n* = 32), with the percentage increase in test score compared between said groups (Fig. 4a). Similar to the findings shown in Fig. 3, we found no significant differences in the percentage increase in test score between these two groups (non-Anki average % increase in test score = 23.8% ± 12.17; Anki average % increase in test score = 21.09% ± 9.08; Fig. 4a). As noted above, however, there were clear differences in the level of usage of Anki in the 32 participants that comprised the Anki group. Therefore, a subsequent analysis was performed wherein four groups were delineated according to the extent of their Anki usage thus: group 1—no engagement with Anki (*n* = 11); group 2—completion of 1–1000 flash cards at the time of the survey (*n* = 11); group 3—completion of 1001–3000 flashcards (*n* = 10); and group 4— completion of 3000–5000 flashcards (*n* = 11). Although there was no significant difference in the percentage increase in test score between those in group 1 and any of the other three groups, there was a significant increase (*P* = 0.0355) in the percentage increase in test score between groups 2 and 4 (Fig. 4b). The mean percentage increase in test score for each Anki usage group was as follows: group 1 (23.81% ± 14.18), group 2 (17.27% ± 7.18), group 3 (21.78% ± 6.12), and group 4 (26.75% ± 11.47). Of note, when the average increase in percentage test score was further broken down by the four individual disciplines assessed in the module, no statistical differences were identified, although group 4 showed a trend towards an increase in the mean score (*P* = 0.0863) for physiology (Fig. 4c).

In order to assess whether demographic factors may have influenced patterns of Anki usage, we examined the distribution of biomedical background, gender, and geographical origin across the four Anki usage groups. This analysis revealed relatively balanced distributions overall, with no obvious demographic bias explaining usage level. While group 1 (non-users) had the highest proportion of female students (91%) and group 4 (highest usage) had a greater proportion of male students (55%), these differences were not formally tested for significance due to sample size limitations. A full breakdown of demographic characteristics by Anki usage group is shown in Table 4.

**Table 4:** Demographic breakdown of participants by Anki usage group. ivided into four groups based on their extent of Anki us. Participants were divided into four groups based on their extent of Anki usage (group 1 = no usage; group 2 = 1–1000 cards; group 3 = 1001–3000 cards; group 4 = 3001–5000 cards). The number and percentage of participants within each group by educational background, gender, and geographic origin are shown

Demographic category	Group 1 (*n* = 11)	Group 2 (*n* = 11)	Group 3 (*n* = 10)	Group 4 (*n* = 11)	Total (*n* = 43)
Biomedical background	9 (81.8%)	9 (81.8%)	8 (80.0%)	10 (90.9%)	36 (83.7%)
Non-biomedical background	2 (18.2%)	2 (18.2%)	2 (20.0%)	1 (9.1%)	7 (16.3%)
Female	10 (90.9%)	8 (72.7%)	5 (50.0%)	5 (45.5%)	28 (65.1%)
Male	1 (9.1%)	3 (27.3%)	5 (50.0%)	6 (54.5%)	15 (34.9%)
North American	9 (81.8%)	7 (63.6%)	7 (70.0%)	8 (72.7%)	31 (72.1%)
EU	2 (18.2%)	4 (36.4%)	3 (30.0%)	3 (27.3%)	12 (27.9%)

### Students’ Perceptions of Usefulness of the Anki App

In addition to gathering quantitative data about the effectiveness of Anki usage, we also sought to determine students’ perceptions of how useful they found the Anki app and specifically why they did or did not use it. This analysis includes all 53 survey responses, including those who did not complete both tests, as it pertains solely to qualitative perceptions rather than assessment performance.

In response to the survey question “Please indicate why you used Anki or why you chose not to use it”, 32/53 responses could be classified as broadly positive regarding their experience and engagement with the Anki app, with the remainder responding to in a negative manner. Those participants who used the Anki app consistently reported liking the “active recall” element and stated that they “know exactly how much I need to review everyday”. Also, this cohort of students focussed on the usefulness of the flashcard system for “keeping material fresh” and “keeping up with last semesters [sic] material”. In addition, several students highlighted that use of this system encouraged them to keep up to date with their learning: “Using Anki forced me to not only study everyday [sic], but to review lectures earlier than I ever would have if I didn’t use the tool”. Many positive comments were contributed, such as “It is a very powerful tool” and “Anki is great!” Within these broadly positive responses, 18 participants commented on the fact that they used the spaced repetition method of learning primarily as a revision, not a learning, tool, as “Anki presents information in a fragmented way” and “doesn’t give me the whole picture”. Thus, these students suggested that once they understood a topic, they found Anki useful for reinforcing the material but not for learning it initially, e.g. “I used Anki as it helped me review things that I had learned weeks prior and not just focusing [sic] on the current content” and “I found it helpful to get reminded of older material”. Of those who chose not to use the Anki spaced repetition-learning tool, students commented on the “stressful” nature of the flashcard system and how it caused them “anxiety”, to feel “guilt” and that it was “overwhelming”. These emotions were associated with the requirement to keep up with the system and to commit to a certain number of cards per day. One student commented that “I think Anki would be very helpful if I found it less stressful to keep up with the daily aspect”.

## Discussion

The main findings of this study were that while 80% of the first year GEM students in UCC who engaged with this study (*n* = 53) reported using Anki, the number of Anki cards used and the daily usage of the technology varied significantly between users. The students’ perceived usefulness of their particular study methodology was generally positive, irrespective of the specific study methodology used. Overall, there was no statistically significant benefit for Anki usage in terms of performance outcome when compared to those who did not use Anki when the module was considered as a whole. However, a significant benefit was noted in two instances: (1) for those who used Anki extensively compared to those who did not engage consistently, and (2) using Anki to study physiology.

These data confirm the importance of personal preference in study methodology among the student body and also the importance of not overemphasising the utility of any particular methodology over another. Specifically, with regard to Anki, several recent reports have emphasised the benefits of spaced repetition learning, particularly in medical curricula [7, 12–14, 23, 24]. Indeed, the creation of an Anki bank comprising 15,000 flashcards by UCC GEM first year students in just over 2 years emphasises the appetite among students for such a spaced repetition learning methodology. This study demonstrates that while certain students respond very well to this mode of learning, and that they value the control that it gives them over their own study progress, there are others who do not find it as useful and even some who respond quite negatively to it. These findings also highlight the fact that a clear time commitment is required to maximise the usefulness of spaced repetition learning, which is not suitable for all students and may be regarded as just another demand on students’ time in what is already a very challenging and overfilled schedule. These insights can serve as important conversation points in academic advising and study skills workshops, helping students to reflect on the alignment between their study methods and their perceived learning outcomes. Educators may consider incorporating such discussions to help students adopt or refine strategies based on their individual preferences and learning goals.

An additional outcome of this study is that it emphasises the importance and value that students place on traditional didactic methods of both teaching and studying. The overwhelming majority of students involved in this study identified lecture attendance as their primary method for learning new material (32/53 respondents) with an additional 10/53 identifying the viewing of recorded lectures as their preferred method for learning new material. This is notable, given that lecture attendance is decreasing with an attendant shift towards more online learning within medical curricula [25]. Similarly, this study also indicates that although many students may use Anki as a study methodology, most view it solely as a tool for reviewing course material and not as a primary method for learning. This was reinforced by the number of comments in the qualitative section of the survey highlighting the use of Anki as a tool for enhancing “recall”. Therefore, students predominantly use Anki as a supplementary learning tool, as a support to both lecture attendance and the creation of their own notes.

Our data also illustrated that Anki use did not significantly improve exam performance relative to those students that did not use the software. This finding is in contrast to previous studies of spaced-repetition learning in medical curricula, which showed that exam performance did improve within groups that utilised spaced-repetition learning [8, 19, 26, 27]. The reason for such a difference in outcome is unclear, but it may be due to the respective methodologies employed. For example, in contrast to the study of Larsen et al. (2009), our study was a broad test of knowledge on material that had been delivered as core content over an entire module, whereas the Larson group utilised a specific interactive teaching session on only two discrete topics [26]*.* It is possible that the improved retention rate found in this study was due to the relatively small amount of content examined. Of note, a recent scoping review regarding the use of electronic flashcards showed that while the majority of studies examining score performance relative to spaced-repetition learning showed increased test scores (11/17 (64%)), 3/17 (18%) found no change, and 3/17 (18%) found changes in some but not all scores [18]. The data generated in this study is therefore in keeping with those that report little score improvement with Anki use [7, 24]. For example, Wothe et al. investigated both academic success and wellness with Anki use in the USA, finding that while there may be some associated benefit with Anki use, it is not a crucial element for success [7]. Our findings demonstrating that Anki usage was associated with a score improvement for physiology alone, therefore, align with the concept that while there may be some benefits, regular Anki usage is not critical for exam performance. Interestingly, our finding that spaced-repetition learning significantly improved the retention of information pertaining specifically to physiology is supported by the work of Dobson, who also found that retrieval practice helped to retain knowledge in physiology [8]. A potential reason for such improvement in physiology with Anki usage is that exam success in physiology relies on critical thinking, which has been shown to be enhanced by spaced repetition learning, as opposed to learning physiology solely than by rote memorisation [2, 28]. It is also possible that the Anki cards prepared for physiology may have been more aptly created, thus benefiting students on UCC exam material.

In the present study, we found a significant increase in average exam performance between cohorts 2 (1–1000 Anki cards) and 4 (3000–5000 Anki cards), suggesting the possibility that those students in cohort 2 represented a category of students that does not engage consistently with any particular study methodology and, indeed, may leave their study efforts until closer to the exam date. This finding is similar to that of Gilbert et al*.* who found that the increase in test scores they observed was most significant for their “high dependency” Anki users [19]. However, although it is tempting to attribute the improvement in test scores for such students as being solely due to increased Anki usage, it is also possible that regular and frequent Anki users may also be the most diligent and committed students that would perform well in exams using any study methodology that they employed.

As there are increasing numbers of students adopting spaced repetition learning methods, there is an onus on faculty to reinforce the importance of trial and error in identifying which are the best study methods for each individual student to adopt. It is clear faculty will need to up-skill in this area in order to better advise and prepare students. Faculty could also become involved in the review and creation of the Anki decks for each module, and in doing so, be better positioned to aid students in use of Anki and identify potential content errors of student-made cards. Faculty could also provide Anki training and support to student cohorts at the start of the academic year to enable informed decisions to be made by student cohorts with respect to learning preferences. Ultimately, it is clear that it will be both necessary and desirable to highlight the variety of study methodologies available to both students and faculty.

### Limitations of the Study

There are various confounding factors to consider. Previous Anki experience may have contributed to improved results in some students. Such students would likely have less time burden associated with initial understanding of the software and would be able to use it more effectively. An additional confounding factor is the previous academic background of students, with those who had previously encountered topics outperforming others to whom the topics are new. These students may be more likely to use recall techniques, as opposed to traditional lecture-based methods for learning new content. Another factor is the time spent on understanding each Anki card. Some students may go through high volumes of cards but not spend time understanding the content. In contrast, others may have less overall daily counts but a richer understanding of material due to increased time spent with interpretation and retention of information. Additionally, one of the study’s limitations is that students, by virtue of having gone through the module, will almost inevitably see an increase in their test scores. Therefore, the reason that we chose to look at the comparative “% increase in scores” between methodologies, rather than the existence of an increase in general, was an attempt to mitigate against this. In our results, pharmacology scores showed little increase between pre- and post-test scores, which might indicate that those questions were particularly challenging. However, the questions were representative of formal end-of-module examination questions. Finally, our study does not account for each student’s studying habits (e.g. hours per week spent studying). This bias could further affect results by indicating which study methodologies in themselves are more time-consuming or require a higher degree of motivation from the learners. For example, the brightest students could be using Anki, as it can be perceived as complicated to utilise.

## Conclusion

The findings of this study suggest that Anki may be a helpful revision tool in preclinical medicine toward medical school examinations, particularly with respect to physiology. Consistent use of Anki proved to be beneficial to perceived knowledge retention. Further research is required to understand the cognitive load of Anki use and its benefits throughout various stages of medical education, including beyond examination scores to practical and clinical applications.

## Supplementary Information

ESM 1 (DOCX 1.53 MB)

## Data Availability

Data is available upon request.
