# Supplementary material for: An Assessment of Anki Flashcards Use in Comparison to Alternative Study Methodologies in First Year Graduate Entry Medical Students
Source: Med Sci Educ. 2025 Oct 6;35(6):2973–83. doi: 10.1007/s40670-025-02504-7 (PMC12961065; doi:10.1007/s40670-025-02504-7)
Supplement: Supplementary file 1 — (DOCX 1.53 MB) [file 40670_2025_2504_MOESM1_ESM.docx]

## Appendix 1

### GEM1 Study Techniques Survey

EVALUATING THE USE OF ANKI AS A STUDY METHODOLOGY AMONG FIRST YEAR GRADUATE ENTRY MEDICAL STUDENTS

*STUDY DESCRIPTION*

Thank you for your time in participating in this study and completing this survey.

A large body of evidence indicates that retrieval practice (test-enhanced learning) and spaced repetition increase long-term information retention. Students may choose to apply such learning strategies when preparing for high-stakes, cumulative assessments, such as the United States Medical Licensing Examination Step 1 and the UCC Graduate Entry to Medicine Programme year 1 exams.

In this study, we wish to investigate whether such a student developed spaced repetition flashcard tool (ANKI) enhances student learning in comparison to other learning strategies. We also wish to study students' perceptions of how useful this tool is in comparison to other traditional learning methodologies.

As you have kindly agreed to take part in this study, you will now have answered both a pre-module set of 40 MCQ questions to enable us to baseline your pre-existing knowledge of the content of the module and then an end of module test of an additional 40 MCQs to gauge your progress with module content. This survey is the final component of the study and will examine your perceptions of the usefulness of ANKI and other learning methodologies.

As the survey tool used, Google forms, does not gather any personal information such as student ID numbers or names, the information you provide will be anonymous. Due to the fact that we are also gathering gender and geographical data however, there is at least a theoretical possibility that individual students could be identified by this information. Whilst this may be a theoretical possibility, we give you our 100% assurance that neither we, nor anyone else involved in this study, would ever seek to identify any individuals who participate in this study. Furthermore, the collected anonymized test and survey information will have no repercussions for any students, individually or as a class, for the modules that the investigator co-ordinates or leads. Thus, in essay type questions please be as forthright as you wish although we would ask that you, please keep your comments constructive.

The survey should take approximately 15 minutes.

As per University regulations,

1. Students must be 18 or over to participate
2. survey participation is voluntary- your participation or any subsequent withdrawal from the study will have no repercussions for you as students or your grades on the GEM Programme.
3. By completing the survey either in full or in part you are indicating that you are voluntarily participating in the study and that any anonymized information provided by you may be analysed and disseminated by the designer of the survey, Dr. Elizabeth Brint.
4. data from the survey will be securely stored for a minimum of 10 years and will then be deleted/destroyed by the designer of the survey Dr. Elizabeth Brint

Section 1: Consent and demographic

The following set of questions in section 1 all pertain to consent and collecting details on participant demographics. Section 2 contains all specific survey questions

1. I consent to take part in this research study having been fully informed of its purpose, and provide my explicit consent to have the anonymous data that I provide to be processed as part of this research study

Mark only one oval.

Yes

No

2. I confirm that I am over 18 years of age

Mark only one oval.

Yes

No

3. In order for us to be able to tally responses for the pre-module test, post-module test and survey data please include below your unique identifier that you used in both pretests consisting of:

1) The 2nd and 5th (in that order) number of your student ID,

2) The 1st number of your birth month, 1st number of your birthday

3) The 3rd, 5th and 7th number of your phone number.

If you did not do the pre-test please now create and enter the above unique identifier such that we can tally survey results with post test data.

4. What age are you as you complete this survey

18-21

22-25

26-29

30+

5. With which gender do you identify?

Female

Male

Other: ___________

6. Please specify which region of the world you originate from?

EU

North America

Other

7. Would you consider your educational background to be biomedical or non-biomedical?

Biomedical

Non-biomedical

Section 2: Experience of study techniques used this term

8. 1. What were your main study methods for **learning new material** this term? Please rank your options by 1 = most often used, and so on.


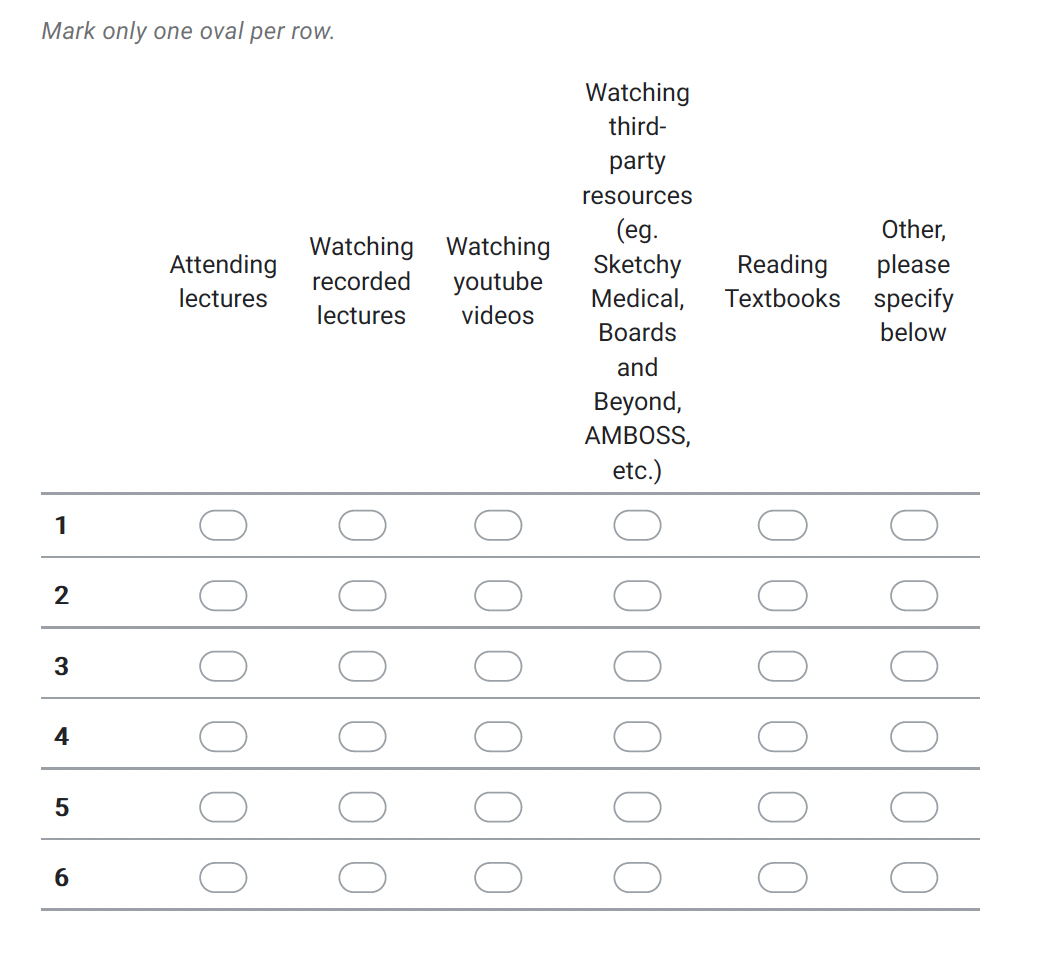


9. 1.a. If you selected other for study methods used to **learn material**, please specify which methods: _________

10. 2. What were your main study methods for **revising and reviewing material** this term? Please rank your options by 1 = most often used, and so on.


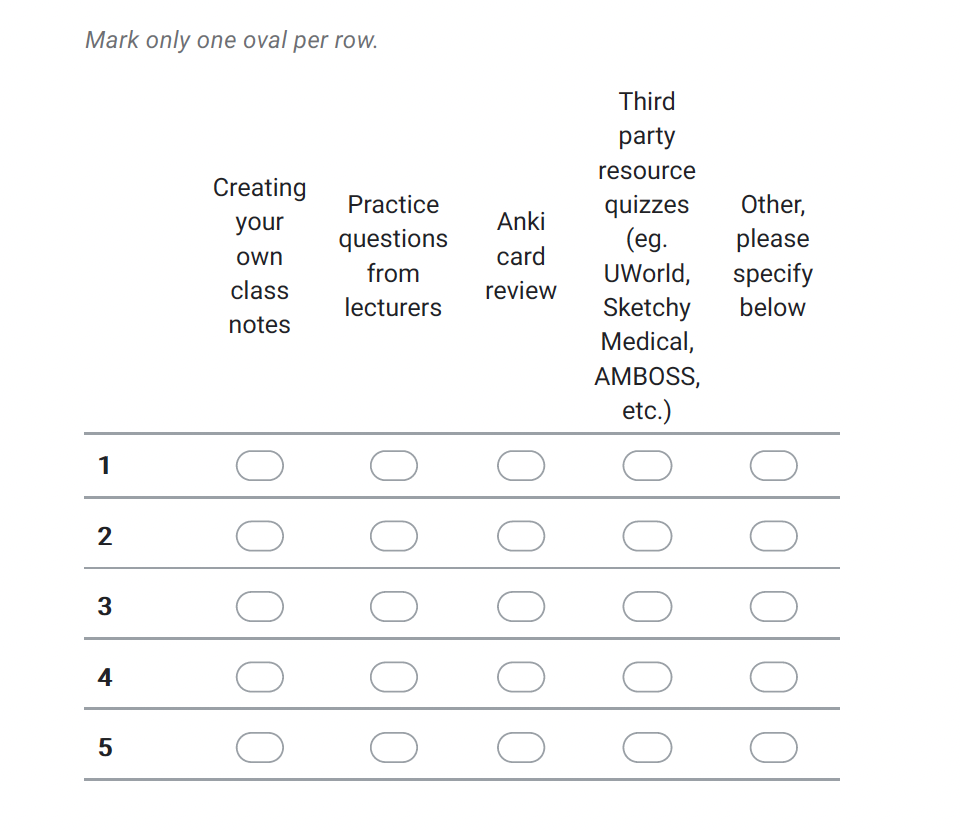


11. 2.b. If you selected other for study methods used **to revise and review material**, please specify which methods:

12. 3. Do you agree with this statement: **I was satisfied with the study techniques I employed this term.**

1 - Strongly Disagree

2 - Disagree

3 - Neither agree nor disagree

4 - Agree

5 - Strongly Agree

13. 4. Did you use Anki this term?

Yes

No - Skip to question 24

14. 5. Please comment on your answer to question 4 – why you used Anki or why you chose not to use it. Please also comment on your experience of the usability of the app.

Section 3: Anki-Related Questions

If you answered "yes" to question 4, please rate your agreement to the following statements:

15. 6. Did you have prior experience using Anki before first year GEM UCC

Yes

No

16. 7. Did you use the Anking deck, Anki Class Deck, or both?

Anking deck

GEM class deck

Both

17. 8. a. How many **mature** cards do you have in your collection?

_______

18. 8. b. Please add a screenshot of your collection stats, as seen in the example below.


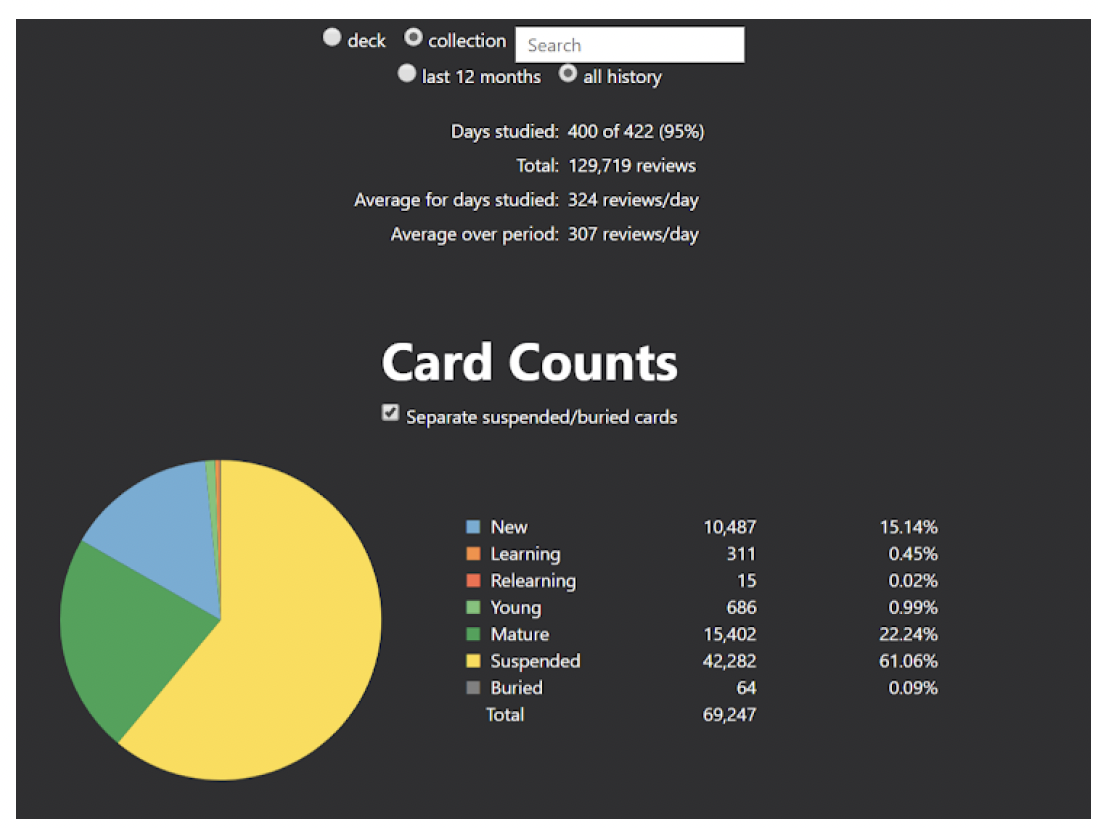


19. 9. a. How many Anki cards have you completed ***per day on average*** over the **last three months**?

__________________

20. 9. b. Please add a screenshot of your collection stats, as seen in the example below.


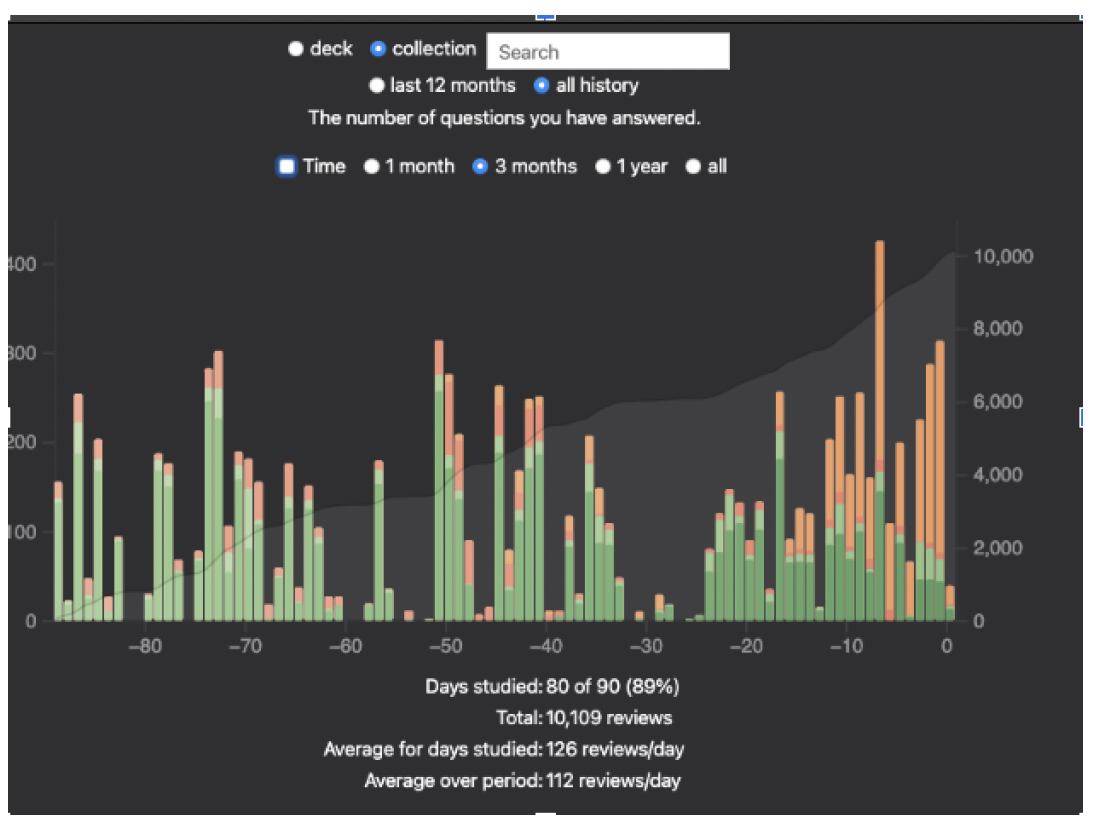


21. 10. **Anki** improved my knowledge and retention of course content during this study period?

1 - Strongly Disagree

2 - Disagree

3 - Neither agree nor disagree

4 - Agree

5 - Strongly Agree

22. 11. I felt adequately prepared for the end of term continuous assessment by using **Anki**

1 - Strongly Disagree

2 - Disagree

3 - Neither agree nor disagree

4 - Agree

5 - Strongly Agree

23. 12. **Anki** allowed me to manage my time in an efficient manner.

1 - Strongly disagree

2 - Disagree

3 - Neither agree nor disagree

4 - Agree

5 - Strongly agree

Section 4: Non Anki-related Questions

If you answered "No" to question 4, or if Anki was not your main study method, please rate your agreement to the following statements:

24. 13. **My study techniques** improved my knowledge and retention of course content during this study period.

1 - Strongly disagree

2 - Disagree

3 - Neither agree nor disagree

4 - Agree

5 - Strongly agree

25. 14. I felt adequately prepared for the end of term continuous assessment by using **my study techniques**

1 - Strongly disagree

2 - Disagree

3 - Neither agree nor disagree

4 - Agree

5 - Strongly agree

26. 15. **My study techniques** allowed me to manage my time in an efficient manner.

1 - Strongly Disagree

2 - Disagree

3 - Neither agree nor disagree

4 - Agree

5 - Strongly Agree

27. 16. When did you started studying for the GM1002 End of Module exam? Eg) 1st day of Term, Last Month, Last week, etc.

_________

28. 18. Do you have any other comments pertaining to your experience with learning

methodologies during the study period?

_________
